# Dual‑Energy CT in Inflammatory Arthritis: A Comprehensive Review

**DOI:** 10.7759/cureus.104695

**Published:** 2026-03-05

**Authors:** Balakrishnan Navaneethakrishnan, Mahabaleshwar Mamadapur, Ponnien Selvan K N

**Affiliations:** 1 Rheumatology, Avinash Hospitals, Chennai, IND; 2 Clinical Immunology and Rheumatology, Jagadguru Sri Shivarathreeshwara (JSS) Medical College, Mysuru, IND; 3 Community Medicine, Government Dharmapuri Medical College, Dharmapuri, IND

**Keywords:** calcium pyrophosphate deposition disease (cppd), crystal arthropathy, dual-energy ct, osteoarthritis, rheumatoid arthritis

## Abstract

Inflammatory arthritis and crystal-associated arthropathies frequently present with overlapping clinical features, creating diagnostic and phenotypic challenges when conventional imaging findings are inconclusive. Dual-energy computed tomography (DECT) extends standard CT by enabling material-specific characterization, allowing differentiation of monosodium urate and calcium-containing deposits, and supporting selected applications beyond crystal detection. This narrative review synthesizes evidence from a curated set of PubMed-indexed studies to examine DECT principles, workflow considerations, interpretation pitfalls, and clinical utility across gout, calcium pyrophosphate deposition disease (CPPD), rheumatoid arthritis (RA), psoriatic arthritis (PsA), axial spondyloarthritis (axSpA), and osteoarthritis (OA). The focus is practical and clinically oriented, emphasizing qualitative evidence synthesis rather than quantitative pooling. DECT has its most established and evidence-supported role in gout, where it provides non-invasive crystal detection, burden mapping, and therapy monitoring. In CPPD, it functions primarily as a problem-solving adjunct when radiographs or ultrasound is inconclusive. In RA and PsA, applications such as iodine overlay imaging and virtual non-calcium reconstruction remain adjunctive and exploratory, with magnetic resonance imaging continuing as the reference standard for inflammatory assessment. In axSpA, DECT strengthens structural staging and may offer selective adjunct marrow evaluation, while in OA, its use is limited to targeted phenotyping in suspected crystal-overlap scenarios. Overall, DECT delivers the greatest clinical value when applied in a disease-specific, question-driven manner within multimodality imaging pathways and interpreted with attention to standardized protocols and potential artifacts.

## Introduction and background

Inflammatory arthritis, including rheumatoid arthritis (RA), psoriatic arthritis (PsA), and axial spondyloarthritis (axSpA), as well as crystal-induced disorders such as gout and calcium pyrophosphate deposition disease (CPPD), frequently presents with overlapping clinical manifestations. Patients commonly report pain, swelling, warmth, and functional limitation, making differentiation based solely on clinical examination challenging in routine practice. Imaging plays a critical role in reducing diagnostic uncertainty by providing objective evidence of inflammation, structural damage, and characteristic deposition patterns. In addition to routine decision-making, imaging supports standardized disease assessment in research and clinical trials. Within this evolving landscape, advanced computed tomography (CT) techniques, particularly dual-energy CT (DECT), are increasingly discussed as part of precision imaging strategies in rheumatology [1].

Even within a single disease entity such as RA, imaging remains indispensable because clinically silent pathological processes, including bone marrow or subchondral alterations, may influence disease classification, prognosis, and treatment monitoring. Accordingly, DECT-based approaches have been explored for visualizing inflammatory features such as bone marrow edema (BME), expanding its role beyond purely structural assessment [2].

Diagnostic overlap becomes especially complex in small joints, particularly in the hands, where crystal arthropathies may imitate or coexist with inflammatory arthritis. High-resolution and material-sensitive imaging techniques are therefore valuable for accurate differentiation and phenotyping [3]. Contemporary reviews describe how DECT is integrated into evolving diagnostic pathways and management algorithms for crystal disease [4]. In clinical rheumatology practice, DECT is increasingly viewed not only as a confirmatory tool for gout but also as a broader problem-solving modality in selected rheumatic conditions [5].

Different inflammatory arthritides exhibit distinct lesion distributions and structural patterns. Multimodality imaging has therefore been emphasized to better characterize differences between RA and PsA when clinical findings overlap [6]. Evidence-based recommendations, including those informing EULAR guidance, reinforce the central role of imaging in crystal-induced arthropathies, particularly when synovial fluid aspiration is not feasible, or presentations are atypical [7].

Imaging is also increasingly used to monitor disease activity and quantify tissue burden. In gout, DECT enables tracking of monosodium urate deposition during therapy, and clinical trial sub-studies have demonstrated its value in assessing treatment response beyond symptom reporting [8]. The technique’s ability to differentiate materials based on energy-dependent attenuation properties explains its particular utility in distinguishing urate from calcium-containing deposits when conventional imaging is equivocal.

Beyond crystal detection, DECT has been investigated for visualization of BME in both injury and inflammatory settings. Meta-analytic evidence supports its performance in detecting lower-limb BME, providing rationale for extending its use to inflammatory arthritis imaging [9]. Broader literature indicates that DECT applications continue to expand, supported by technical advances in acquisition and reconstruction, including material decomposition maps, virtual non-calcium (VNCa) images, and iodine overlays [10, 11].

In axial disease, systematic reviews have evaluated DECT accuracy for sacroiliac joint BME detection, which is relevant when MRI is limited or structural assessment is prioritized [12]. DECT has also been explored for extra-articular urate deposition, including vascular tissues, highlighting its ability to assess disease burden beyond joints [13]. General musculoskeletal reviews position DECT as a multipurpose imaging tool while emphasizing challenges such as standardization and artifact interpretation that require careful consideration [14,15].

In gout, DECT has shown potential prognostic value, correlating imaging findings with clinical outcomes [16]. Systematic reviews further support VNCa applications for non-traumatic BME detection [17]. In axSpA, low-dose CT and DECT are discussed as evolving tools, particularly when MRI access is constrained [18].

Material differentiation remains the defining contribution of DECT in crystal arthropathies such as gout and CPPD [19]. Evidence from acute knee injury imaging demonstrates how DECT adds tissue characterization beyond conventional CT [20]. Reviews on chondrocalcinosis and CPPD reinforce its role when radiography is inconclusive [21], while multiple gout-focused analyses detail its contribution to disease mapping and burden assessment [22].

VNCa techniques illustrate the flexibility of DECT outputs across clinical questions [23], and trauma-oriented literature supports its broader interpretive framework [24]. Comparative discussions position DECT as complementary to ultrasound in crystal disease management rather than a replacement modality [25]. Clinical utility reviews in gout consolidate its diagnostic and monitoring roles [26].

Emergency imaging reviews further show how DECT extends CT from structural to selective functional assessment [27]. Multimodality comparisons in inflammatory arthritis support integrating DECT alongside MRI and ultrasound for specific clinical questions [28]. Broader CT discussions in rheumatology frame DECT within advanced imaging pathways aligned with precision medicine principles [29,30].

Diagnostic certainty in crystal arthritis still relies on appropriate reference standards, especially synovial fluid analysis. Literature consistently positions DECT as an additive rather than a complete substitute for aspiration [31]. Investigations of extra-articular urate deposition [32], meta-analyses of gout diagnostic accuracy [33], and systematic reviews addressing diagnosis and follow-up [34] strengthen its evidence base. However, artifact-related false positives and protocol variability underscore the importance of standardized interpretation [35].

Overall, DECT has evolved from a crystal-detection tool into a broader, question-driven imaging modality with selective applications across inflammatory and degenerative arthropathies.

Aim and scope of this review

The evidence base informing this review includes disease-specific investigations and broader musculoskeletal DECT applications. Studies evaluating CPPD deposit detection in the knee supported focused discussion of CPPD imaging [36,37], while musculoskeletal reviews enable structured coverage across rheumatologic diagnoses [38]. Comparative analyses contrasting CT and MRI perspectives reinforce the importance of clarifying where DECT complements rather than replaces conventional modalities [39].

This review synthesizes evidence using a structured framework addressing four practical dimensions: clinical utility (diagnosis, phenotyping, and monitoring); potential substitution of conventional pathways (radiography, ultrasound, MRI, and synovial aspiration); workflow considerations; and overall evidence strength. In gout, discussion is grounded in disease-focused reviews highlighting real-world utility and interpretive limitations [40]. For “beyond crystal” applications, evidence supporting VNCa techniques is incorporated to contextualize BME visualization within inflammatory arthritis imaging [41].

The gout section integrates diagnostic accuracy studies, clinical impact analyses, and Cureus-published experience [42-44]. In PsA, iodine overlay findings are discussed alongside comparative MRI studies to clarify performance and limitations [45-47]. Structural and periarticular involvement, including tendon and ligament deposits, is addressed where evidence supports comprehensive burden mapping [48].

The review concludes with a cross-disease synthesis summarizing DECT targets, clinical roles, replacement potential, workflow implications, and evidence strength across gout, CPPD, RA, PsA, axSpA, and osteoarthritis, providing a practical reference framework for contemporary rheumatologic imaging practice [49,50].

## Review

Study design

This article was designed as a narrative, clinically oriented review examining the role of DECT in inflammatory arthritis and crystal-associated arthropathies. The emphasis is practical and application-driven, focusing on clinical utility, interpretive considerations, and workflow integration rather than quantitative synthesis.

The literature was derived from a curated reference set limited to 50 PubMed-indexed articles. PubMed was searched using combinations of keywords including “dual-energy CT,” “DECT,” “gout,” “CPPD,” “rheumatoid arthritis,” “psoriatic arthritis,” “axial spondyloarthritis,” “bone marrow edema,” and “virtual non-calcium.” Priority was given to systematic reviews, meta-analyses, comparative imaging studies, and disease-specific clinical investigations published predominantly between 2016 and 2025. Articles were considered eligible if they informed one or more predefined thematic domains relevant to clinical implementation, interpretation, or decision-making. These domains included DECT principles and technical foundations, reconstruction techniques and workflow, artifact recognition, modality comparison, disease-specific applications, and emerging “beyond crystal” roles.

No additional databases were searched, and no formal risk-of-bias assessment was performed, as this work was structured as a qualitative narrative review. Findings were synthesized descriptively, and no quantitative pooling or meta-analysis was conducted. The evidence was organized into structured thematic sections addressing DECT fundamentals, workflow considerations, modality comparisons, and disease-specific applications. Each disease section evaluates clinical utility, potential substitution of conventional imaging pathways, workflow implications, and overall evidence strength. The review concludes with a cross-disease comparative synthesis to facilitate practical interpretation. Because no new human or animal data were collected, institutional review board approval and informed consent were not required.

DECT fundamentals

DECT acquires imaging data using two distinct X-ray energy spectra. Because materials attenuate X-rays differently at varying energy levels, DECT applies material decomposition algorithms to distinguish substances that may appear similar on conventional single-energy CT. The processed output can be displayed as color-coded overlays on grayscale CT images, allowing simultaneous structural evaluation and material characterization [11,14].

In rheumatologic imaging, this capability extends CT beyond structural depiction toward material-specific assessment. DECT can differentiate monosodium urate from calcium-containing deposits and can demonstrate iodine distribution following contrast administration. However, accurate interpretation depends on appropriate acquisition parameters, standardized post-processing techniques, and awareness of potential artifacts that may influence image appearance [15].

Common DECT reconstructions and output maps

In musculoskeletal practice, DECT generates different reconstruction outputs depending on the clinical question. These include monosodium urate maps displayed as color overlays for identification and quantification of urate deposition, calcium-focused reconstructions highlighting calcific deposits, iodine overlay maps following contrast administration to depict inflammatory enhancement, and VNCa images that suppress trabecular calcium signals to improve visualization of edema-like marrow changes.

Material differentiation forms the foundation of DECT application in gout and CPPD [19]. Urate mapping contributes to diagnostic confirmation and assessment of disease burden in gout [22], while VNCa reconstructions expand the role of DECT beyond crystal detection into marrow-related evaluation [23].

Practical workflow for DECT in rheumatology

A DECT examination follows a structured workflow that integrates conventional CT acquisition with dual-energy-specific post-processing. The process begins with defining the clinical indication, followed by careful patient positioning to minimize motion and optimize region-of-interest alignment. Dual-energy acquisition is then performed, after which standard grayscale reconstructions are generated for anatomical assessment. Material-specific post-processing subsequently produces targeted maps such as urate overlays, calcium maps, iodine maps when contrast is used, and VNCa reconstructions.

Interpretation requires careful correlation of material overlays with grayscale CT images to identify artifacts and avoid misclassification. Final reporting should integrate structural findings with material-specific information and include an explicit statement regarding diagnostic confidence and relevant limitations. DECT is most effective when applied within a question-driven framework and interpreted alongside clinical context and complementary imaging modalities [29,30]. The structured workflow of DECT acquisition, reconstruction, post-processing, and reporting is summarized in Table 1.

**Table 1:** DECT workflow, outputs, and reporting checklist *Iodine overlays require contrast-enhanced DECT. DECT: Dual-energy computed tomography

Step	What happens	Output	Practical reporting note
1. Clinical question	Define the indication (e.g., suspected gout vs CPPD vs inflammatory activity vs BME question).	Protocol selection	Clearly state the clinical question in the report, as it determines which DECT maps are generated [29,30].
2. Patient positioning	Align the region of interest; minimize motion; remove avoidable external contaminants.	Improved data quality	Document any quality limitations (motion, limited coverage, metal artifacts) that may reduce diagnostic confidence [29,30].
3. Dual-energy acquisition	Acquire the same anatomy at two energy spectra (scanner-dependent implementation). If iodine mapping is intended, contrast administration is required*.	Dual-energy dataset	Record anatomical coverage, laterality, and whether intravenous contrast was used (particularly if iodine maps are generated) [29,30].
4. Reconstruction	Generate standard grayscale CT reconstructions.	Conventional CT images	Describe structural findings (e.g., erosions, osteophytes, calcifications) alongside material maps [29,30].
5. Material post-processing	Apply material decomposition to generate targeted maps (e.g., urate, calcium, VNCa, iodine*).	Color overlays / material maps	Specify which material maps were analyzed (MSU, calcium, VNCa, iodine*) and interpret them within anatomical context [30].
6. Interpretation	Correlate overlays with grayscale CT; assess for artifacts before concluding “true deposits” or inflammation.	Integrated interpretation	Explicitly mention artifacts when present and adjust diagnostic certainty if needed [30,35].
7. Final report	Summarize deposits, inflammation, and structural findings, including limitations and clinical implications.	Actionable impression	Provide a confidence statement and recommend next steps if findings are discordant with the clinical picture [29,30,35].

Artifacts and interpretation pitfalls

The reliability of DECT depends not only on its technical capabilities but also on careful interpretation of material-specific overlays. False-positive findings may arise if artifacts are not recognized and correlated with grayscale images [35]. Nail-related keratin artifacts may produce urate-like signals at superficial locations, while patient motion can cause misregistration between the two energy datasets, resulting in displaced or patchy overlays. Beam hardening from dense structures or metallic hardware may distort spectral separation and create misleading signals. Dense calcifications may generate spillover effects at their margins, and partial-volume phenomena in small anatomical regions may produce isolated “hot spots.” VNCa reconstructions may be influenced by parameter variability or underlying sclerosis, and iodine overlays may reflect non-specific hyperemia rather than disease-specific inflammation.

Material overlays should always be interpreted in conjunction with grayscale CT images and clinical findings. Signals located near skin surfaces or at the edges of the field of view should be approached cautiously. When artifacts are suspected, they should be clearly documented in the radiology report, and diagnostic confidence should be adjusted accordingly [35,37]. Advanced reconstructions such as VNCa and iodine mapping require disciplined acquisition and standardized interpretation to minimize the risk of overcalling inflammatory or marrow-related abnormalities (Table 2) [41,42].

**Table 2:** Common DECT artifacts/pitfalls and mitigation Closing link to the broader DECT foundation: These fundamentals—principle, workflow, and artifact-aware interpretation—are consistent with established musculoskeletal DECT frameworks used for crystal disease and broader MSK applications [50]. DECT: Dual-energy computed tomography

Artifact/pitfall	Appearance	Why it occurs	Mitigation	Clinical impact
Keratin/nail (urate false positive)	Small focal urate‑colored signals near nails/skin	Material misclassification at keratin/skin interfaces	Correlate with grayscale CT; avoid overcalling superficial/edge signals	False gout diagnosis/overestimated urate burden [35,37]
Motion/misregistration	Patchy, displaced, or “floating” overlay	Misalignment between low-/high-energy datasets	Immobilization; repeat scan if severe; interpret with caution	False positives/negatives; reduced confidence [35]
Beam hardening	Streaks with a spurious overlay signal	High attenuation distorts spectral separation	Optimize acquisition; iterative recon where available; interpret streak regions cautiously	Misleading deposits/inflammation; interpretive uncertainty [35]
Metal hardware	Streaking, noisy maps, irregular overlays	Photon starvation/scatter → decomposition errors	Metal artifact reduction (if available); limit region; consider alternate modality if nondiagnostic	Reduced diagnostic value; may obscure true findings [35]
“Spillover” around dense structures	Color overlay around dense calcification/edges	Partial‑volume + thresholding effects	Confirm on grayscale CT in multiple planes; avoid interpreting edge artifacts as deposits	Crystal‑type confusion; false positives [35]
Partial volume (small anatomy)	Tiny “hot spots” at boundaries	Mixed tissues within one voxel	Thin reconstructions; multi‑planar review; interpret tiny isolated signals cautiously	Overcalling very small deposits [35]
VNCa pitfalls (BME over/under‑calling)	Apparent edema‑like signal that may not match the clinical picture	Parameter dependence; confounding sclerosis/technical factors	Interpret with clinical correlation; avoid using as MRI substitute without context	Misclassification of inflammatory activity/lesion burden [41]
Iodine overlay pitfalls	Apparent enhancement pattern not specific to one diagnosis	Contrast timing/technique dependence; non‑specific hyperemia	Standardize protocol; correlate with other imaging/clinical findings	Overinterpretation of “inflammation” without specificity [42]

How DECT differs from radiography, conventional CT, MRI, and ultrasound

Plain radiography remains the foundational imaging modality in rheumatology, particularly for assessing structural damage such as erosions, joint space narrowing, and chondrocalcinosis in CPPD [19,30,39]. It is widely available and cost-effective but lacks sensitivity in early disease and provides limited soft-tissue or inflammatory assessment.

Conventional single-energy CT offers superior spatial resolution for evaluating bone erosions, cortical defects, and calcifications [39,50]. However, it does not provide material decomposition and cannot reliably differentiate urate from calcium deposits.

MRI is the primary modality for assessing inflammatory activity, including synovitis and bone marrow edema (BME) [19,39]. Its strength lies in soft-tissue and marrow visualization without ionizing radiation. However, MRI cannot directly perform material differentiation and therefore cannot distinguish urate from calcium deposits with the specificity of DECT.

Ultrasound is widely used in crystal disease evaluation due to its real-time capability and Doppler assessment of inflammation [25,30,37]. It is accessible and dynamic but operator-dependent and limited in deep or complex anatomical regions.

DECT uniquely combines structural CT details with material-specific characterization, positioning it as a complementary problem-solving tool rather than a universal replacement modality, as presented in Table 3.

**Table 3:** Comparison of DECT with radiography, conventional CT, MRI, and ultrasound DECT: Dual-energy computed tomography; BME: bone marrow edema

Modality	Best detects	Strengths	Limitations	Typical use	Time (relative)	Cost (relative)	Replace? (Y/N/Selective)
DECT	Material‑specific deposits (urate vs calcium); plus structural CT detail [19,50]	Unique material decomposition with color overlays; strong for crystal characterization; combines with CT anatomy [19,50]	Artifacts can mimic deposits; needs appropriate post‑processing and expertise [30,50]	Problem‑solving for crystal disease; mapping burden; adjunct in complex cases [19,30,50]	Short–moderate [50]	Moderate–High [50]	Selective (can replace other tests in some scenarios, but usually complements) [19,30]
X‑ray	Structural damage, calcifications/chondrocalcinosis [19,30,39]	Fast, available, low cost; good baseline structure/calcification overview [19,39]	Limited sensitivity in early disease; poor soft‑tissue inflammation detail; no material typing [19,30,39]	First‑line baseline imaging; structural staging; chondrocalcinosis screening [19,30,39]	Very short [39]	Low [39]	No (often first-line but insufficient alone in many scenarios) [19,30]
Conventional CT (single‑energy)	Bone anatomy, erosions, dense calcifications [39,50]	Excellent spatial resolution; strong structural assessment [39,50]	Lacks DECT material separation for urate vs calcium; radiation consideration [39,50]	Structural problem‑solving when anatomy detail is needed [39,50]	Short [50]	Moderate [50]	Selective (may replace X‑ray for structure in select cases; not a crystal-typing tool) [39,50]
MRI	Synovitis, BME, soft‑tissue inflammation [19,39]	Best for inflammatory activity and soft tissues; no ionizing radiation [19,39]	Longer exam time; access/cost; limited for direct crystal material typing [19,39]	Inflammatory arthritis activity assessment; early disease; marrow/soft tissue questions [19,39]	Long [39]	High [39]	No (gold standard for some inflammation questions but not for material typing) [19,39]
Ultrasound	Crystal signs + Doppler hyperemia; superficial soft tissues [25,30,37]	Bedside, dynamic, Doppler activity; useful in crystal disease workups [25,30,37]	Operator dependence; limited in deep joints/complex anatomy; variable reproducibility [25,37]	First-line adjunct in crystal disease and inflammatory assessment; guided aspiration [25,30,37]	Short [25]	Low–Moderate [25]	Selective (can substitute for some assessments; often complementary with DECT) [25,37]

Gout

DECT has its most established and evidence-supported role in the evaluation of gout. By applying material decomposition algorithms, it enables non-invasive identification of monosodium urate (MSU) deposits, which are displayed as color-coded overlays aligned with conventional grayscale CT images [22,25,26,33,34,44,49]. This material-specific capability provides objective confirmation of crystal deposition and strengthens diagnostic confidence, particularly in complex clinical scenarios.

DECT is especially useful when joint aspiration is not feasible, technically difficult, or yields inconclusive results. It also adds value in patients with atypical clinical presentations or when overlap with other inflammatory arthritides complicates diagnosis. Beyond confirming the presence of urate crystals, DECT allows mapping of urate distribution across multiple joints and periarticular structures, thereby supporting disease phenotyping and estimation of total urate burden [22,26,48,50]. Serial DECT examinations can further be used to monitor crystal depletion during urate-lowering therapy, complementing clinical assessment and biochemical markers such as serum urate levels [8,16,26]. Emerging evidence indicates that quantitative assessment of total urate burden may carry prognostic significance and influence therapeutic decision-making [16,47] (Table 4).

**Table 4:** Role of DECT in gout: clinical scenarios and influence on management decisions DECT: Dual-energy computed tomography; MSU: monosodium urate

Clinical scenario (when DECT is considered)	What DECT adds (decision impact)	Key limitations to note	Refs
Suspected gout where aspiration is not feasible/declined/non‑diagnostic	Provides objective evidence of MSU deposition to support diagnosis	Artifacts can mimic urate → must correlate with grayscale CT	[26,35,43]
Atypical presentation or overlap with other arthritides	Helps distinguish gout by mapping MSU deposits and distribution patterns	Not a stand‑alone substitute when imaging/clinical mismatch is strong	[22,25,26,49]
Mapping burden in established gout (e.g., tophaceous disease)	Visualizes and maps burden across joints and periarticular tissues	Requires consistent protocol for follow‑up comparison	[22,26,48,50]
Monitoring urate‑lowering therapy/crystal depletion	Tracks changes in MSU burden over time; supports monitoring strategies	Interpretation should account for artifacts and protocol differences	[8,16,26]
When ultrasound is limited (deep joints/complex anatomy; operator dependence issues)	Provides less operator‑dependent, anatomically comprehensive assessment	Radiation and availability considerations	[25,26,37,50]
When radiographs are negative but suspicion remains high	Detects MSU deposition that may precede clear radiographic damage	X‑ray still useful for structural staging; DECT targets deposits	[25,26,49]

Although DECT can support diagnosis when synovial fluid analysis cannot be performed, it should not replace aspiration when feasible, as fluid analysis remains an important reference standard [25,26,35,49]. Ultrasound continues to provide practical bedside assessment, and radiography retains a role in structural staging. Accordingly, DECT functions primarily as a selective adjunct within a multimodality framework rather than as a universal substitute.

From a workflow perspective, DECT acquisition is relatively brief; however, post-processing and artifact evaluation require appropriate expertise. While it is generally more costly than radiography and often ultrasound, it is typically less time-consuming than MRI and can be efficiently integrated into clinical practice when standardized protocols are established [5,26,50].

The evidence base for DECT in gout is robust. Multiple systematic reviews and meta-analyses demonstrate strong diagnostic performance for MSU detection [33,34,44], and clinical impact studies suggest that DECT findings may influence management decisions in patients with suspected gout [47]. Overall, gout represents the most well-supported and clearly defined clinical indication for DECT in rheumatologic imaging are detailed in Table 5.

**Table 5:** Evidence base for DECT in gout: findings from meta-analyses and studies of clinical outcomes and monitoring DECT: Dual-energy computed tomography

Evidence category	Focus of analysis	Clinically relevant conclusion	Key references
Meta-analysis	Accuracy of DECT in diagnosing gout	DECT shows strong diagnostic performance for detecting monosodium urate deposition when applied in appropriate clinical scenarios	[33,44]
Systematic review	Role of DECT in diagnosis and longitudinal assessment	Evidence supports the use of DECT for both diagnostic confirmation and follow-up, provided that standardized acquisition and interpretation protocols are followed	[34]
Systematic literature review (added/prognostic value)	Incremental value of DECT beyond clinical assessment	DECT contributes additional information related to crystal burden and potential prognostic insight beyond routine clinical evaluation	[16]
Trial-associated imaging evidence	Assessment of crystal burden changes during therapy	DECT-based endpoints can demonstrate reductions in urate deposition during treatment, supporting its utility in therapy monitoring	[8]
Clinical impact study	Influence of DECT on management decisions and outcomes	Incorporation of DECT into diagnostic pathways may alter treatment strategies and influence clinical outcomes in patients with suspected gout	[47]
Interpretation and safety considerations	Recognition and management of imaging artifacts	Reliable DECT interpretation in gout requires awareness of common artifacts (e.g., keratin- or nail-related false positives) to preserve diagnostic confidence	[35]

Calcium pyrophosphate deposition disease

CPPD can clinically resemble gout, rheumatoid arthritis flares, or exacerbations of osteoarthritis, which may create diagnostic uncertainty in routine practice. Although radiography can demonstrate chondrocalcinosis, its sensitivity is limited, particularly in early disease or when deposits are subtle, deeply located, or confined to complex intra-articular structures [19,21,30].

In this context, DECT enables visualization of calcium-containing deposits within menisci, articular cartilage, and ligaments, most commonly in the knee joint [19,36]. It is particularly helpful when radiographic findings are equivocal, when synovial fluid aspiration is not feasible or inconclusive, or when diagnostic uncertainty persists despite conventional imaging. By improving the depiction of deposits in fibrocartilaginous and deep intra-articular structures that are difficult to assess with radiography or ultrasound, DECT can enhance diagnostic confidence and support more accurate phenotyping.

Within the diagnostic pathway, DECT should be regarded as a complementary problem-solving modality rather than a first-line investigation. Plain radiography continues to serve as the initial imaging tool, and ultrasound may assist in evaluating superficial joints. DECT adds value primarily when conventional imaging findings do not adequately explain the clinical picture or when there is persistent suspicion of CPPD despite non-diagnostic baseline studies [19,21,30]. The principal clinical scenarios and decision points for DECT use in CPPD are summarized in Table 6.

**Table 6:** CPPD clinical utility and indications for ordering DECT Source: [19,21,30,36,50] DECT: Dual-energy computed tomography; CPPD: calcium pyrophosphate deposition disease

Clinical scenario	Added value of DECT	Role within the diagnostic pathway	Practical consideration
Acute flare with unclear differentiation between gout, CPPD, or other inflammatory arthritis	Assists in supporting CPPD by visualizing calcium-containing crystal deposition in disease-relevant structures	Complements clinical evaluation and radiography; useful when synovial aspiration is not feasible	Particularly helpful when radiographs are negative or equivocal despite persistent suspicion
Suspected CPPD involving complex or deep structures (e.g., knee meniscus)	Enables visualization of deposits within menisci or cartilage that may be difficult to characterize on plain radiographs	Adds diagnostic information beyond radiography and can address limitations of ultrasound in deep anatomy	A joint-focused DECT protocol is often sufficient
Equivocal chondrocalcinosis on radiographs	Provides additional clarification regarding the presence and distribution of calcific deposits	Radiography remains the baseline modality; DECT functions as a problem-solving tool	Findings should be interpreted alongside symptoms and structural imaging
Recurrent flares with persistent diagnostic uncertainty	Supports more confident classification of the underlying crystal phenotype	Complements longitudinal clinical assessment and conventional imaging findings	Structured reporting with acknowledgment of confidence and limitations is recommended

The evidence supporting DECT in CPPD includes focused detection studies, such as investigations demonstrating its ability to identify meniscal CPPD deposits, as well as narrative and systematic reviews discussing advances in chondrocalcinosis imaging [21,36]. However, compared with gout, the volume and strength of evidence are more limited, and large prospective outcome-based studies remain lacking. A concise overview of supporting evidence categories is presented in Table 7.

**Table 7:** CPPD: summary of supporting evidence DECT: Dual-energy computed tomography; CPPD: calcium pyrophosphate deposition disease

Evidence category	Focus of study	Clinically relevant conclusion	Key references
Original detection study	Identification of CPPD crystal deposition in the knee meniscus using DECT	Demonstrates the feasibility and clinical usefulness of DECT for detecting fibrocartilaginous CPPD deposits	[36]
Review (diagnostic imaging advances)	Imaging strategies for chondrocalcinosis and CPPD	Emphasizes the role of advanced imaging when radiography provides limited diagnostic information	[21]
Review (crystal disease framework)	Comparative imaging pathways across crystal-associated arthropathies	Positions DECT as an adjunct and problem-solving modality alongside radiography and ultrasound	[19,30]

Rheumatoid arthritis

In contrast to gout and CPPD, where DECT is primarily applied for crystal detection, its role in RA centers on adjunctive assessment of inflammation and structural damage. One of the explored applications is contrast-enhanced DECT with iodine overlay mapping, which can depict synovial enhancement patterns associated with inflammatory activity [42]. These iodine maps have been investigated as potential imaging surrogates for synovitis; however, they are not currently regarded as primary diagnostic standards and should be interpreted within a broader multimodality context.

In addition, VNCa reconstructions have been evaluated for their ability to demonstrate BME in RA [2,17,23,41]. By suppressing the trabecular calcium signal, VNCa techniques aim to improve visualization of edema-like marrow changes. Although conceptually promising, this application remains exploratory and does not replace MRI, which continues to be the reference standard for marrow assessment. The principal DECT applications in RA, including iodine mapping, structural evaluation, and VNCa-based marrow assessment, are summarized in Table 8.

**Table 8:** DECT applications in rheumatoid arthritis: synovitis mapping, structural evaluation, and bone marrow edema assessment DECT: Dual-energy computed tomography; BME: bone marrow edema

DECT application in RA	What it shows (output)	Clinical utility	Does not replace	Key References
Iodine overlay/iodine mapping	Color-coded iodine enhancement pattern highlighting areas of inflammatory activity	Adjunct for synovitis evaluation; useful when MRI access is limited or contraindicated	MRI for comprehensive inflammation assessment	[42]
Structural assessment (CT component of DECT)	High-resolution depiction of bones, erosions, and structural anatomy	Detailed structural evaluation when precise bone-level information is needed	MRI for marrow and soft tissue inflammation	[5,6,28]
Virtual non-calcium (BME-focused) approach	Edema-like signal generated by suppressing trabecular calcium contributions	Exploratory adjunct for marrow-related questions; useful in selected research or clinical scenarios	MRI remains the primary tool for BME assessment	[2,17,23,41]

Beyond inflammatory assessment, DECT retains the structural advantages of conventional CT, allowing high-resolution depiction of erosions, cortical defects, and osseous architecture. This may be particularly helpful when MRI is unavailable, contraindicated, or when precise bone-level evaluation is required [5,6,28]. Nonetheless, DECT should be positioned as an adjunct rather than a substitute for established imaging modalities in RA. MRI remains central for evaluation of synovitis and BME, and ultrasound continues to provide valuable information in inflammatory joint assessment [6,28]. Contrast-enhanced DECT may offer supplementary information in selected scenarios, particularly when MRI access is limited [42].

The evidence base supporting DECT in RA includes clinical feasibility studies of iodine overlay imaging [42], observational investigations assessing VNCa reconstructions for BME [2], and systematic reviews that provide methodological support for virtual non-calcium applications [17,41]. However, compared with gout, the available data are more limited, and current evidence should be considered emerging rather than definitive. A structured summary of the supporting evidence categories is presented in Table 9.

**Table 9:** Evidence summary of DECT applications in rheumatoid arthritis DECT: Dual-energy computed tomography; RA: rheumatoid arthritis; BME: bone marrow edema; PsA: psoriatic arthritis

Evidence type	Focus of evaluation	Clinical takeaway	Key references
Original clinical study	Iodine overlay DECT for inflammatory arthritis imaging	Confirms the feasibility of iodine mapping to visualize inflammatory activity; supports adjunctive synovitis assessment	[42]
Observational study	DECT evaluation of bone marrow edema in RA	Demonstrates exploratory use of DECT for BME; supports the “beyond crystals” concept	[2]
Systematic review/meta-analysis	Diagnostic performance of DECT for non-traumatic BME	Provides evidence for virtual non-calcium approach; underpins adjunctive use in inflammatory assessment	[17,41]
Technical/clinical methods review	Virtual non-calcium DECT applications	Explains generation and interpretation of VNCa outputs and potential clinical applications	[23]
Comparative imaging context	Lesion differences in RA vs PsA	Reinforces that DECT is complementary and should be integrated with multimodality imaging strategies	[6,28]
Practice guidance	DECT in rheumatology	Positions DECT within real-world rheumatology imaging workflows	[5]

Psoriatic arthritis

PsA frequently demonstrates overlapping clinical and imaging features with rheumatoid arthritis, particularly in the small joints of the hands. Because patterns of joint involvement and inflammatory distribution may differ between these conditions, accurate differentiation is important for guiding management decisions, and multimodality imaging is often required [6,28].

In PsA, the main DECT application that has been explored is contrast-enhanced iodine overlay imaging. These iodine maps depict enhancement patterns within joints and periarticular tissues, reflecting inflammatory activity [45]. By integrating structural CT detail with color-coded iodine overlays, DECT enables simultaneous evaluation of osseous anatomy and enhancement distribution, which may assist in structured assessment of disease involvement. The key clinical scenarios in which DECT may be considered in PsA are outlined in Table 10.

**Table 10:** Psoriatic arthritis DECT utility and key clinical questions DECT: Dual-energy computed tomography; RA: rheumatoid arthritis; PsA: psoriatic arthritis

Ideal clinical question in PsA	DECT output	Clinical utility	Recommended comparator/pairing	Key references
Can inflammatory activity in hand PsA be mapped using CT-based imaging?	Iodine overlay/iodine map (contrast DECT)	Highlights enhancement patterns reflecting inflammatory activity in affected joints	MRI remains the core standard for inflammatory assessment	[45]
Are lesion patterns more consistent with RA vs PsA when clinical findings overlap?	CT anatomy ± iodine overlay adjunct	Supports problem-solving in multimodality imaging for differentiating lesion patterns	Multimodality imaging emphasized for RA vs PsA differentiation	[6,28]
MRI is limited — can DECT provide adjunct inflammatory information quickly?	Iodine overlay (if contrast used) + CT structural context	Serves as a potential adjunct when MRI is inaccessible or contraindicated	Should be interpreted within a multimodality framework; not a universal replacement	[6,28,45]

From a clinical standpoint, DECT should be regarded as a selective adjunct rather than a primary imaging modality in PsA. MRI remains the principal technique for evaluating synovitis and bone marrow edema. However, DECT may provide additional information in selected situations, particularly when MRI is unavailable or contraindicated, when CT is already being performed for another indication, or when further clarification of inflammatory distribution is required. Comparative studies have demonstrated the feasibility of iodine overlay imaging in PsA but continue to reinforce MRI’s central role in comprehensive inflammatory assessment [45].

The supporting evidence for DECT in PsA is currently limited and consists mainly of comparative imaging studies and feasibility investigations, along with broader multimodality discussions addressing differentiation between RA and PsA [6,28,45]. A concise summary of the available evidence categories is presented in Table 11.

**Table 11:** Psoriatic arthritis evidence summary with comparative imaging focus DECT: Dual-energy computed tomography; RA: rheumatoid arthritis; PsA: psoriatic arthritis

Evidence type	Study focus	Main clinical conclusion	Key references
Comparative imaging study	Contrast-enhanced DECT iodine overlay in hand PsA vs contrast MRI	Confirms feasibility of DECT iodine overlay as an adjunct tool for inflammation mapping in selected contexts	[45]
Multimodality imaging context	Imaging features used to differentiate RA vs PsA (hand-focused)	Reinforces the importance of integrated multimodal imaging for accurate diagnosis	[28]
Pattern-focused inflammatory lesion discussion	Differences in inflammatory lesion patterns between RA and PsA	Supports careful imaging selection when clinical overlap exists	[6]
Practice framing	DECT applications in rheumatology and musculoskeletal imaging	Provides context for workflow and implementation of DECT in routine clinical practice	[5,50]

Axial spondyloarthritis

In axSpA, imaging primarily addresses two objectives: identifying active inflammatory changes and evaluating structural damage.MRI remains the preferred modality for detecting active inflammation, whereas CT provides superior depiction of structural abnormalities.

Within this context, DECT retains the high spatial resolution of conventional CT and is well suited for assessing sacroiliac joint erosions, subchondral sclerosis, and ankylosis [1,18]. Its structural details may assist in disease staging and in resolving diagnostically challenging cases where anatomy is complex or conventional imaging is inconclusive. Beyond structural evaluation, VNCa reconstructions have been investigated for the detection of bone marrow edema at the sacroiliac joints [12,23]. By suppressing the trabecular calcium signal, VNCa techniques aim to improve visualization of edema-like marrow changes. Meta-analytic data support a potential adjunctive role for this approach; however, MRI continues to serve as the reference standard for assessment of active inflammatory lesions [12]. The clinical questions and corresponding roles of DECT in axSpA are summarized in Table 12.

**Table 12:** Axial spondyloarthritis: DECT role in structural and inflammatory assessment DECT: Dual-energy computed tomography; BME: bone marrow edema; VNCa: virtual non-calcium

Clinical question in axSpA	Best primary modality	DECT utility	Can DECT replace?	Key references
Is there active inflammation (e.g., bone marrow edema)?	MRI	Serves as an adjunct or investigational tool; evidence supports evaluation of SIJ BME using VNCa	No, MRI remains standard	[12,18,23]
What is the structural stage (erosions, sclerosis, ankylosis)?	CT	Maintains CT’s structural resolution; supports staging, problem-solving, and trial imaging	Selective; may outperform radiographs	[1,18]
MRI is contraindicated or unavailable — what alternatives exist?	CT-based pathway	DECT supports targeted structural evaluation within CT workflow; interpretation must consider limitations	Selective	[1,18]
Can low-dose CT/DECT be integrated efficiently?	CT (low-dose strategies)	Low-dose protocols enhance workflow efficiency compared with MRI; feasible for clinical use	Not a universal replacement	[18,50]

From a clinical perspective, DECT should be considered complementary rather than substitutive. It may be particularly relevant when MRI is contraindicated, unavailable, or when the clinical priority is detailed structural evaluation [18]. The use of low-dose CT protocols may further improve feasibility and integration into practice while maintaining attention to radiation considerations [18]. The available evidence base consists of review articles discussing the evolving role of DECT and low-dose CT in axSpA [18], a systematic review and meta-analysis evaluating DECT for sacroiliac joint bone marrow edema [12], and technical reviews describing the principles and applications of VNCa imaging [23]. Overall, while promising, the evidence remains emerging and does not yet support routine replacement of MRI for inflammatory assessment. A summary of key evidence sources is provided in Table 13.

**Table 13:** Axial spondyloarthritis: evidence summary (reviews and meta-analyses) DECT: Dual-energy computed tomography; BME: bone marrow edema; VNCa: virtual non-calcium

Evidence type	Focus of evaluation	Key clinical conclusions	Key references
Review (axSpA-specific)	Future applications of low-dose CT and DECT in axSpA	Positions DECT and low-dose CT as evolving tools for structural assessment and selected adjunct uses	[18]
Review (broader inflammatory arthritis CT)	Advanced CT applications in inflammatory arthritis clinics and trials	Demonstrates how CT and advanced CT approaches can integrate into clinical and trial workflows	[1]
Systematic review/meta-analysis	Accuracy of DECT for detecting BME in sacroiliac joints	Provides an evidence base supporting VNCa/BME evaluation as an adjunct in axial disease	[12]
Technical methods review	Clinical applications of virtual non-calcium DECT	Describes the VNCa concept and framework for assessing marrow signal changes	[23]
Musculoskeletal DECT overview	General implementation of DECT in musculoskeletal imaging	Supports workflow, feasibility, and interpretation of DECT outputs in clinical practice	[50]

Osteoarthritis

OA is a common degenerative joint disorder that may coexist with crystal-associated conditions, particularly CPPD. In such situations, differentiating age-related degenerative calcifications from crystal-related deposits can be clinically difficult, especially when imaging findings and symptom severity do not fully correlate [19,21,39].

DECT does not have a routine role in standard OA staging. Its potential contribution is primarily in selected scenarios where additional clarification is required. This includes cases with suspected crystal overlap, evaluation of deep or anatomically complex calcifications, or situations in which patient symptoms appear disproportionate to radiographic findings. By combining high-resolution structural imaging with calcium-specific material mapping, DECT may assist in more refined phenotyping when degenerative and crystal-related processes coexist [14,19,50]. The specific clinical contexts in which DECT may be considered in OA are summarized in Table 14.

**Table 14:** Osteoarthritis: DECT appropriateness, contribution, and complementary imaging DECT: Dual-energy computed tomography; OA: osteoarthritis

Clinical scenario/question	DECT contribution	Complementary modality/typical pathway	Key references
OA patient with suspected calcification overlap or chondrocalcinosis	Determines presence and distribution of calcium deposits; supports overlap phenotyping	Complements radiography and clinical evaluation; aids diagnostic clarification	[19,21]
Episodic flare-like symptoms disproportionate to radiographic OA	Provides problem-solving information when X-rays are equivocal or insufficient	Used after baseline radiographs; may complement ultrasound depending on setting	[19,21]
Complex anatomy or deep structures (e.g., knee fibrocartilage)	Offers CT-level structural detail plus material-specific visualization of calcifications	Complements radiography; applied selectively for detailed assessment	[19,50]
Research or phenotyping context (selected centers)	Enables higher-resolution phenotyping for OA with calcification overlap	Typically used in research; not routine clinical staging	[14,50]

From a practical standpoint, conventional radiography remains the first-line imaging modality for OA assessment. DECT should be reserved for targeted problem-solving when baseline imaging and clinical evaluation do not provide sufficient explanation. The available evidence supporting DECT in OA is limited and largely derived from broader reviews and conceptual discussions addressing crystal overlap. There are currently no large prospective OA-specific outcome studies that justify the routine use of DECT in degenerative joint disease. A summary of the supporting evidence and existing gaps is presented in Table 15.

**Table 15:** Osteoarthritis: DECT evidence summary and research gaps DECT: Dual-energy computed tomography; OA: osteoarthritis

Evidence type	Contribution to OA discussion	Key support	Remaining gaps/limitations	Key references
Crystal disorder imaging reviews	Contextualizes OA symptoms in relation to crystal overlap and calcification	Provides rationale for selective DECT use in suspected overlap	Limited prospective OA-specific studies evaluating DECT outcomes	[19,21]
CT/MRI perspectives in crystalline arthropathies	Clarifies the roles of CT and MRI in differential diagnosis	Reinforces DECT as an adjunct rather than a replacement for standard OA imaging	Insufficient evidence supporting DECT as a replacement for radiography	[39]
Musculoskeletal DECT overviews	Offers technical and clinical guidance for DECT as a phenotyping tool	Supports feasibility and map-based interpretation in musculoskeletal imaging	Lack of standardized OA-overlap protocols and validated clinical impact pathways	[14,50]

Beyond crystals (cross-cutting evidence)

Beyond its established role in crystal detection, DECT has been explored for additional applications that extend into marrow and inflammatory assessment. One of the most discussed approaches is VNCa imaging, in which suppression of the trabecular calcium signal enhances the visibility of edema-like marrow changes [23,41]. This technique has been evaluated in both traumatic and non-traumatic settings, and systematic reviews as well as meta-analyses have reported supportive diagnostic performance for detecting BME in selected contexts [9,17,20]. These findings provide a conceptual basis for considering VNCa as an adjunct in inflammatory arthritis imaging. However, interpretation requires caution, as reconstruction parameters, underlying sclerosis, and technical variability may influence signal appearance. For this reason, VNCa should be regarded as a complementary technique rather than a replacement for MRI in marrow evaluation. Experience from emergency and trauma imaging further illustrates how VNCa can broaden the utility of CT in acute settings when MRI is unavailable or delayed [24,27]. A structured overview of cross-cutting DECT applications, including VNCa, is provided in Table 16.

**Table 16:** Evidence map for “beyond crystals” DECT applications DECT: Dual-energy computed tomography; BME: bone marrow edema; RA: rheumatoid arthritis; PsA: psoriatic arthritis

Beyond-crystal DECT application	Output type	Main evidence context in your set	What it supports clinically	Key refs
Virtual non-calcium (VNCa) for BME	VNCa reconstructions (calcium-suppressed images)	Methodological + clinical performance work across multiple BME scenarios	Positions BME depiction as an adjunct CT output that can add marrow-level information	[23,41,17]
BME detection (injury settings)	VNCa-type reconstructions	Systematic review/meta-analysis in acute knee injury; meta-analysis in lower-limb injury	Supports CT-based marrow characterization when MRI is unavailable, delayed, or impractical	[20,9]
Emergency implementation of DECT for BME	VNCa integrated into ED workflows	Emergency radiology/ED-oriented overview (Radiographics)	Demonstrates workflow feasibility and potential value of adding marrow assessment to CT in acute care	[27]
Trauma-focused DECT applications	DECT recon outputs (including VNCa)	Trauma-oriented DECT review	Provides clinical plausibility and interpretation context for VNCa-type outputs in trauma pathways	[24]
Iodine overlay for inflammatory activity	Iodine mapping/overlay (contrast-enhanced DECT)	Inflammatory arthritis applications; framed within RA vs PsA discussion	Supports adjunct depiction of enhancement patterns consistent with inflammatory activity	[42,6]
Iodine overlay vs MRI comparison	Iodine overlay contrasted with MR enhancement	Hand PsA study comparing DECT iodine overlay with contrast-enhanced MRI	Supports “comparative role vs MRI” framing in selected contexts	[45]

Another expanding application involves iodine mapping following contrast-enhanced DECT acquisition. Iodine overlay images demonstrate contrast distribution within tissues, reflecting hyperemia and increased vascular permeability, and may therefore assist in visualizing inflammatory activity [42]. In inflammatory arthritis, including hand PsA, iodine overlay imaging has been directly compared with contrast-enhanced MRI and shown to offer feasible adjunctive information [45]. Nevertheless, MRI continues to serve as the reference standard for comprehensive inflammatory lesion assessment. Accordingly, iodine mapping should be positioned within a multimodality framework as a selective, question-driven adjunct rather than as an independent inflammatory imaging tool [6,42,45].

Costs, time, and implementation

The successful integration of DECT into clinical practice depends not only on the availability of dual-energy-capable scanners but also on structured workflow, trained personnel, and careful interpretation. DECT may be understood as comprising two interconnected components: conventional CT acquisition with standard reconstruction, followed by dual-energy-specific post-processing that generates material maps for clinical interpretation. While scan acquisition is generally brief, the additional steps of material decomposition, artifact assessment, and structured reporting require dedicated expertise and familiarity with technique-specific limitations [11].

DECT is most effective when applied to clearly defined clinical questions, such as confirmation of crystal deposition or focused diagnostic clarification. Standardized acquisition protocols and reporting frameworks enhance reproducibility and consistency across institutions [5,50]. Because DECT involves ionizing radiation, attention to dose optimization remains essential, particularly in axial imaging where larger anatomical regions may be examined and follow-up imaging may be necessary [18]. Practical implementation therefore varies across centers and depends not only on hardware capability but also on institutional workflow maturity and experience.

Final cross-disease comparison

Across rheumatologic conditions, the strength and maturity of evidence supporting DECT vary considerably. Its most robust and well-validated indication remains gout, where multiple systematic reviews and meta-analyses demonstrate strong diagnostic performance for detection of monosodium urate deposition [33,34,44]. In this setting, DECT provides non-invasive confirmation, mapping of crystal burden, and support for therapy monitoring.

In CPPD, DECT plays a more selective role. It may assist in identifying calcium-containing deposits when radiographs are inconclusive, particularly in complex intra-articular locations. Available evidence supports a complementary, problem-solving function rather than routine application [19,21,36].

In RA and PsA, DECT applications primarily involve iodine overlay techniques for inflammation mapping and virtual non-calcium methods for marrow assessment. Although feasibility has been demonstrated, current data remain exploratory, and MRI continues to be central for comprehensive inflammatory evaluation [2,42,45].

In axSpA, DECT offers clear value in structural staging due to its high spatial resolution and may provide adjunctive marrow assessment through VNCa techniques. Nevertheless, MRI remains the standard for detecting active inflammatory lesions [12,18].

In osteoarthritis, the role of DECT is limited and largely confined to selected cases with suspected crystal overlap. Current evidence does not support its routine use in degenerative joint disease [14,19]. A consolidated comparison of DECT applications across conditions is presented in Table 17.

**Table 17:** DECT applications across rheumatologic and musculoskeletal conditions (Time and cost reflect relative tiers for clinical pathway planning, not absolute measures.) DECT: Dual-energy computed tomography; CPPD: calcium pyrophosphate deposition disease; RA: rheumatoid arthritis; PsA: psoriatic arthritis; axSpA: axial spondyloarthritis; OA: osteoarthritis; BME: bone marrow edema; VNCa: virtual non-calcium

Disease	Primary DECT target (MSU/Calcium/Iodine/vNC-BME)	Main clinical utility	Replacement potential (No/selective/often)	Time (relative)	Cost (relative)	Evidence strength	Key references
Gout	MSU	Non-invasive MSU detection; burden mapping; therapy monitoring; problem-solving when aspiration not feasible	Selective	Short	Moderate	High	[22,25,26,33,34,35,44,49]
CPPD	Calcium	Detection and characterization of CPPD deposits in meniscus/cartilage; clarifies diagnosis when X-ray is equivocal	Selective	Short	Moderate	Moderate	[19,21,30,36]
RA	Iodine (synovitis) + vNC-BME (exploratory)	Adjunct synovitis mapping (iodine overlay); investigational BME assessment; CT-level structural evaluation	Selective	Moderate	Moderate–High	Emerging	[2,5,17,23,41,42]
PsA	Iodine	Adjunct inflammation mapping in selected scenarios; comparative role vs MRI in hand PsA	Selective	Moderate	Moderate–High	Limited	[5,6,28,45]
axSpA	Structural (SI joints) + vNC-BME (adjunct concept)	Structural staging and problem-solving; potential adjunct framework for SIJ BME evaluation; low-dose DECT strategies	Selective	Short–Moderate	Moderate	Emerging	[1,12,18,23]
OA	Calcium (overlap phenotyping)	Problem-solving for calcification overlap (OA + CPPD); selective phenotyping rather than routine staging	No	Short	Moderate	Limited	[14,19,21,39,50]

Limitations and pitfalls

A major limitation of DECT, particularly in crystal mapping workflows, is the potential for artifacts and false-positive findings that may be misinterpreted as true deposits. Recognizing common artifacts, such as superficial or edge signals, motion-related misregistration, and beam hardening effects, is critical to maintain diagnostic accuracy and avoid overestimation of disease burden [35].

DECT is not a single, uniform technique; its outputs depend heavily on the acquisition protocol, reconstruction methods, and post-processing algorithms used to generate material-specific maps. Differences in these technical parameters can affect map appearance and interpretability, leading to variability across centers if standardized protocols are not implemented [11,14].

Although DECT has a robust evidence base in gout, applications in inflammatory arthritis, such as iodine overlays for synovitis or VNCa for bone marrow edema, and degenerative disease phenotyping remain less well validated. These areas often rely on limited outcome-based studies and are highly dependent on standardized methods and trained interpretation for reliable clinical use [11,14,35].

Future directions

Ongoing research is expected to refine the integration of advanced CT techniques, including DECT, into both routine clinical care and clinical trials. Emphasis will be placed on standardized acquisition methods and clinically relevant endpoints. Reviews on advanced CT in inflammatory arthritis underscore the importance of this integration for improving both diagnostic workflows and trial outcomes [1].

Broader frameworks for CT in rheumatology highlight the potential for quantitative and advanced CT-based techniques to play an increasing role within imaging pathways. These developments may support more precise disease assessment and enhance multimodality decision-making [29].

Low-dose CT and DECT strategies are particularly relevant in axial spondyloarthritis, where imaging often requires coverage of larger anatomical regions and may be repeated over time. Future research emphasizes dose optimization as a critical factor for broader clinical adoption [18].

Establishing standardized imaging protocols, reproducible post-processing workflows, structured reporting, and multicenter validation is essential to ensure that DECT-derived findings are reliable and comparable. This is particularly important for emerging “beyond crystal” applications, where clinical evidence is still evolving [1,29].

## Conclusions

DECT has an established and clearly evidence-supported role in the evaluation of gout. It enables non-invasive detection and mapping of monosodium urate deposits and allows objective assessment of crystal burden, including monitoring during urate-lowering therapy. In this setting, the strength of available systematic reviews and meta-analyses supports its integration into selected diagnostic pathways when clinically indicated. In CPPD, DECT serves primarily as a complementary tool. It may assist in clarifying calcium deposition when radiographs are inconclusive or when there is persistent diagnostic uncertainty, but it does not replace conventional first-line imaging or synovial fluid analysis. In RA, PsA, and axSpA, DECT currently functions as a problem-solving modality rather than a primary imaging technique. Applications such as iodine-based inflammation mapping and VNCa reconstruction for marrow assessment are promising but remain adjunctive. MRI continues to be the reference standard for evaluation of active inflammation, and ultrasound retains an important role in clinical practice. In osteoarthritis, the role of DECT is limited and should be reserved for selected cases with suspected crystal overlap or unresolved diagnostic questions. Current evidence does not support routine use in degenerative disease staging. Overall, DECT provides the greatest clinical value when applied in a disease-specific, question-driven manner and integrated thoughtfully within multimodality imaging pathways. Its interpretation requires standardized protocols, awareness of artifacts, and appropriate clinical correlation. With continued refinement and validation, DECT may further strengthen its role in precision imaging within rheumatology.
